# Simultaneous pharmacokinetic and pharmacodynamic analysis of 5α-reductase inhibitors and androgens by liquid chromatography tandem mass spectrometry

**DOI:** 10.1016/j.talanta.2014.07.087

**Published:** 2015-01

**Authors:** Rita Upreti, Gregorio Naredo, Abdullah M.M. Faqehi, Katherine A. Hughes, Laurence H. Stewart, Brian R. Walker, Natalie Z.M. Homer, Ruth Andrew

**Affiliations:** a Endocrinology, University/British Heart Foundation Centre for Cardiovascular Science, Queen׳s Medical Research Institute, University of Edinburgh, 47, Little France Crescent, Edinburgh EH16 4TJ, United Kingdom; b Mass Spectrometry Core, Wellcome Trust Clinical Research Facility, Queen׳s Medical Research Institute, University of Edinburgh, 47, Little France Crescent, Edinburgh EH16 4TJ, United Kingdom; c Department of Urology, NHS Lothian, Western General Hospital, Crewe Road South, Edinburgh EH4 2XU, United Kingdom

**Keywords:** BPH, benign prostatic hyperplasia, Testosterone, Dihydrotestosterone, 5α-reductase, Dutasteride, Finasteride, Liquid chromatography tandem mass spectrometry

## Abstract

Benign prostatic hyperplasia and prostate cancer can be treated with the 5α-reductase inhibitors, finasteride and dutasteride, when pharmacodynamic biomarkers are useful in assessing response. A novel method was developed to measure the substrates and products of 5α-reductases (testosterone, 5α-dihydrotestosterone (DHT), androstenedione) and finasteride and dutasteride simultaneously by liquid chromatography tandem mass spectrometry, using an ABSciex QTRAP^®^ 5500, with a Waters Acquity™ UPLC. Analytes were extracted from serum (500 µL) via solid-phase extraction (Oasis^®^ HLB), with ^13^C_3_-labelled androgens and d9-finasteride included as internal standards. Analytes were separated on a Kinetex C18 column (150×3 mm, 2.6 µm), using a gradient run of 19 min. Temporal resolution of analytes from naturally occurring isomers and mass +2 isotopomers was ensured. Protonated molecular ions were detected in atmospheric pressure chemical ionisation mode and source conditions optimised for DHT, the least abundant analyte. Multiple reaction monitoring was performed as follows: testosterone (*m*/*z* 289→97), DHT (*m*/*z* 291→255), androstenedione (*m*/*z* 287→97), dutasteride (*m*/*z* 529→461), finasteride (*m*/*z* 373→317). Validation parameters (intra- and inter-assay precision and accuracy, linearity, limits of quantitation) were within acceptable ranges and biological extracts were stable for 28 days. Finally the method was employed in men treated with finasteride or dutasteride; levels of DHT were lowered by both drugs and furthermore the substrate concentrations increased.

## Highlights

• Simultaneous analysis of 5α-reductase inhibitors with enzyme substrates and products.
• Combined pharmacokinetic/pharmacodynamic analysis.
• Rapid, sensitive and robust analysis of androgens, dutasteride and finasteride.

## Graphical abstract

**Figure:**
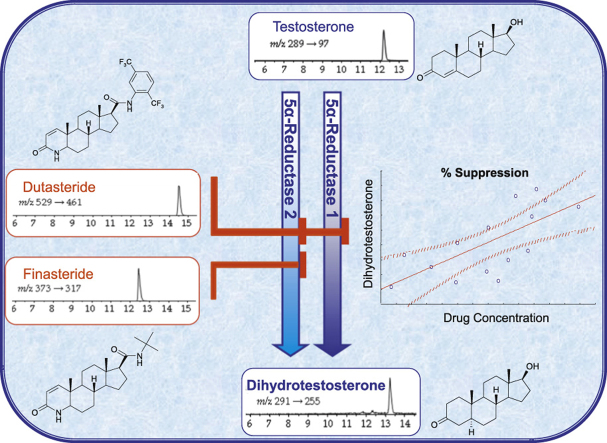

## 1 Introduction

**Fig. 1:**
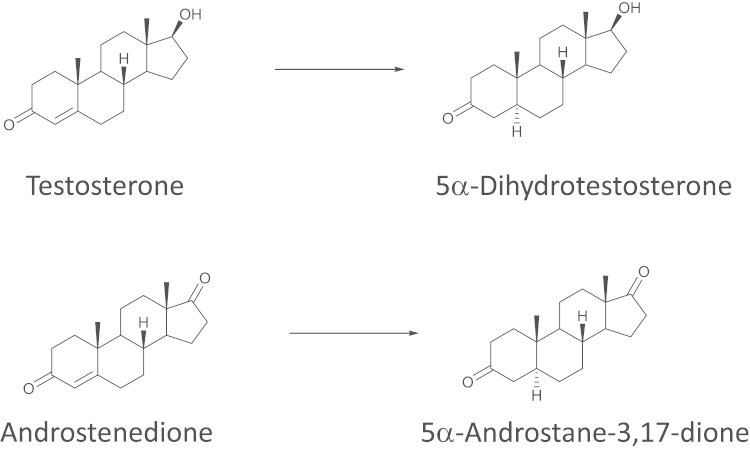
Reactions catalysed by 5α-reductases 1 and 2. 5α-Reductases catalyse the irreversible reduction of the 4-5 double bond in the A-ring, e.g. of testosterone and androstenedione.

Finasteride and dutasteride, are irreversible inhibitors of 5α-reductase isozyme(s) [1]. They were developed to decrease the conversion of testosterone to its more potent metabolite 5α-dihydrotestosterone (DHT) (Fig. 1) in the treatment of benign prostatic hyperplasia (BPH) and are now being proposed for use in prostate cancer [2]. In its early stages, prostate cancer is androgen-responsive and androgen ablation therapy is effective in restraining tumour growth [3]. As the disease advances, the tumour becomes “castration resistant”, with changes in the responsiveness of the androgen receptor (AR) and in its associated signalling pathways. Under these circumstances, local androgen synthesis inhibitors, such as 5α-reductase inhibitors, may be used to further lower levels of any remaining intra-tumoural androgen [4], often derived from adrenal sources. In many therapeutic settings where 5α-reductase inhibitors are used or studied, the simultaneous assessment of pharmacodynamics and pharmacokinetics is desirable, and best achieved by measurement of steroid concentrations and drug concentrations respectively.

Finasteride, the first drug in class, is selective for 5α-reductase type 2 [5], exerting its effects most markedly in the prostate. Dutasteride is a “dual” 5α-reductase type 1 and 2 inhibitor [6], developed to lower the levels of DHT further, achieving ~94% reduction in DHT compared with ~70–80% suppression by finasteride [7], [8], [9], [10]. However, by inhibiting 5α-reductase 1, it has the potential to influence the hormonal milieu in a wider array of tissues [11], notably the liver. 5α-Reductases metabolise not only androgens but also glucocorticoids, mineralocorticoids and progestogens. Therefore, inhibitors of 5α-reductase isozymes, and in particular of type 1 5α-reductase may have effects on diverse steroid hormone signalling pathways. Recent studies suggest that inhibition of 5α-reductase type 1 in liver may adversely influence insulin sensitivity [12], predispose to fatty liver [13] and also alter stress responses [14].

Pharmacodynamic and pharmacokinetic studies of 5α-reductases are achieved by measurement of the androgenic substrate and product, together with circulating drug concentrations. Such measures also permit assessment of treatment adherence in clinical studies. To minimise sample volume, maximise efficiency of sample processing, allow analysis without unblinding participants, and permit simultaneous pharmacodynamic and pharmacokinetic evaluation, a single assay measuring both drugs and androgen concentrations is desirable. Dutasteride [15], [16] and finasteride [17], [18], [19], [20], [21], [22] have been quantified previously by liquid chromatography–mass spectrometry (LC–MS), but only individually. Testosterone and androstenedione, the principle endogenous androgenic substrates of 5α-reductase circulate in ~1–30 nM concentrations [23], [24], and testosterone is routinely monitored by LC tandem MS ( LC–MS/MS) in clinical biochemistry laboratories [25]. However, analysis of DHT presents particular challenges due to its low concentrations, especially following 5α-reductase inhibition [8] and poor propensity to ionise. The use of LC tandem MS (LC–MS/MS) has allowed measurement of DHT in adult [24] and paediatric populations [26], overcoming the need for derivatisation with GC–MS methods [27], [28], the low sensitivity and lack of mass separation with HPLC methods with ultraviolet detection [29] and the lack of specificity with immunoassays [30]. While many approaches to quantify DHT by LC–MS have also required derivatisation [31], [32], [33], this may not be possible in conjunction with simultaneous analysis of drug levels.

We developed a novel assay simultaneously measuring inhibitors (finasteride and dutasteride), substrates (testosterone, androstenedione) and product (DHT) of 5α-reductases in human serum. The approach was evaluated in monitoring pharmacodynamic responses to 5α-reductase inhibitors in men.

## 2 Materials and methods

### 2.1 Reagents and standards

Unless stated otherwise, chemicals (including 2,3,4-[^13^C_3_] labelled androgens) were from Sigma-Aldrich (Dorset, UK) and all solvents were from Rathburn Chemical Ltd (Walkerburn, UK). Water and formic acid (FA) were from Fisher Scientific (Loughborough, UK). Methanol (HPLC gradient grade) was from VWR (Lutterworth, Leicestershire, UK). Finasteride was from Steraloids (Newport, RI, USA) and dutasteride from AK Scientific (Mountain View, CA, USA). 23,23,23,24,24,24,25,25,25[^2^H_9_]-finasteride (d9-finasteride) was synthesised in-house [34]. Stock solutions were prepared at 1 and 0.01 mg/mL in methanol, and stored at −20 °C. Working solutions were prepared on the day of analysis.

### 2.2 Biological samples

Pooled male human serum and steroid-stripped serum for the method of optimisation and validation were from TCS Biosciences (Buckingham, UK). Due to residual androstenedione and testosterone being detectable in the steroid-stripped serum, it was re-stripped before use. Dextran-coated charcoal was added to steroid-stripped serum (0.1 g/10 mL), stirred (~24 h, 4 °C) and removed by centrifugation (1811*g*, 4 °C, 30 min). Stripped serum was sequentially filtered through 1.20 µm (Sartorius minisart, Sartorius AG, Göttingen, Germany) and 0.22 µm filters (Millex^®^ GP filter unit, Millipore Ireland Ltd., Carrigtwohill, Ireland) until clear and aliquots frozen (−20 °C) until use.

### 2.3 LC–MS/MS instrumentation and MS tuning

Chromatographic separation was performed on a Waters Acquity™ UPLC (Manchester, UK) with autosampler, and detection on an ABSciex QTRAP® 5500 mass spectrometer (Warrington, UK), operated with Analyst® Software version 1.5.1. Nitrogen was the source, curtain and collision gas. Compound specific tuning (collision energy, cell exit potential and declustering potential) in positive atmospheric pressure chemical ionisation (APCI) mode was performed using methanolic solutions of steroids, internal standards and drugs. The masses of precursor ions were determined and transitions yielding the most abundant product ions selected from the eight most abundant transitions screened. MS source conditions were then optimised for DHT, the least abundant analyte; final optimised conditions were curtain gas 25 psi, collision gas low, spray voltage 5 kV, nebuliser current 3.5 µA, source temperature 500 °C, and ion source gas 55 psi.

### 2.4 Extraction and chromatographic method

Samples were extracted via solid-phase extraction (Oasis® HLB, 30 μm, 30 mg (Waters, Elstree, UK)). Extraction cartridges were primed with methanol (1 mL) then water (1 mL). Samples (500 µL), enriched with internal standard (1 ng), were mixed with water (500 µL) and loaded onto primed extraction cartridges. After a wash step (50% methanol in water, 1 mL), analytes were eluted with methanol (1 mL). Eluates were dried under oxygen-free nitrogen (37 °C) and reconstituted in mobile phase (30:70 methanol:water+0.1% FA; 100 µL). Injection volume was 10 µL.

Analytes were eluted at 35 °C from a Kinetex C18 column (150×3 mm, 2.6 µm, Phenomenex^®^, Macclesfield, UK) with a 1 min hold followed by a 9 min linear gradient from 30:70 to 80:20 (methanol:water with 0.1% FA) at a flow rate of 250 µL/min. Conditions were sustained until 16 min followed by re-equilibration.

## 3 Assay validation

### 3.1 Recovery

Recovery was calculated by expressing the mean of the integrated peak areas from extracted standards as a percentage of that of unextracted standards. This was performed in replicates of 6 using stripped serum as matrix and enriched with androstenedione (1 ng), testosterone (1 ng), DHT (10 ng), finasteride (1 ng) and dutasteride (10 ng).

### 3.2 Ion suppression by serum

The effect of the biological matrix (human serum) on ionisation efficiency was assessed in replicates of 6 by post-spiking extracted blank serum with all analytes in amounts corresponding to the midpoint of the standard curve (indicated by ⁎), and responses compared with those of standards with the same amounts of analytes dissolved in mobile phase (30:70 methanol:water +0.1% FA). Blank serum sample were also analysed so amounts of endogenous analytes could be subtracted from peak areas detected in post-spiked samples.

### 3.3 Analyte specificity

Extracts of blank stripped and unstripped serum were analysed and checked for interferences at or close to the expected retention times for androgens and drugs and internal standards respectively. Chromatographic resolution was ensured between anticipated endogenous stereo or positional isomers, e.g., testosterone, epi-testosterone and dehydroepiandrosterone (DHEA), and also between endogenous hormones and predicted mass +2 isotopomeric interferents, e.g., mass +2 testosterone and DHT. Ratios of quantifier and qualifier mass transitions were monitored for analytes all except DHT, where the ion generated by the qualifier transition was not detected in serum. Quantifier:qualifier ratios in biological samples were considered acceptable if within 20% of the mean ratio of standards.

### 3.4 Limits of detection (LOD) and lower limits of quantitation (LLOQ)

LOD were determined by analysing solutions prepared by serial dilution of analyte and internal standard stock solutions, with the LOD assigned to a peak area where signal:noise ratio (SNR) was ≈3. LLOQ following extraction was determined by extracting analyte and internal standard from serum at amounts corresponding to 0.2×LOD, 0.5×LOD, LOD and 5/3×LOD. The LLOQ was defined as the amount where relative standard deviation in replicates of 6 was ≤20%.

### 3.5 Linearity

Two standard curves were generated, one in water (for quantitation of androgens) and one in serum (for quantitation of dutasteride and finasteride), with 1 ng of each internal standard. Standard curves represented concentration ranges: testosterone (1, 2^#^, 3, 5, 7.5^,#^, 10⁎, 12.5, 15^#^ ng/mL), androstenedione and DHT (0.1, 0.2^#^, 0.5, 1, 2^⁎^^,#^, 3, 4, 5^#^ ng/mL), finasteride and dutasteride (1, 2^#^, 5, 10, 25^⁎^^,#^, 50, 75, 100^#^ ng/mL); ^⁎^ used to assess ion suppression, ^#^ used to assess accuracy and precision. Peak areas of each analyte and internal standard were integrated and a calibration curve constructed (peak area ratio of analyte/internal standard versus concentration of analyte). Regression lines of best fit were constructed and considered acceptable if the regression coefficient, *r*, was >0.99. Accuracy was compared using different weightings (none, 1/*x* and 1/*x*^2^).

### 3.6 Precision and accuracy

The intra-assay accuracy and precision were determined with 3 points of the standard curve prepared in replicates of 6 (low, medium, high, indicated by # above). Precision was also determined using 6 replicates of a patient sample. The inter-assay accuracy and precision were determined from four standard curves prepared on different days. The precision was calculated as the relative standard deviation of the mean (RSD) with RSD (%)=standard deviation/mean×100. The % accuracy was calculated as the calculated concentration/ theoretical concentration×100. Injector variability was assessed by injecting (6 times) the midpoint standards (medium) of the calibration curve, and a pooled male serum sample enriched with both finasteride (10 ng/mL) and dutasteride (20 ng/mL).

### 3.7 Stability

Stability was assessed by reinjection of a single calibration curve and patient sample after 24 h in the auto-sampler (10 °C) and following 28 day storage (−20 °C). Acceptable storage conditions were those giving ≤10% change in response.

### 3.8 Method exemplification

Drugs and steroids were quantified in serum collected from male subjects (age 20–85 years, *n*=16/group) prior to and following 90 days of treatment once daily with either dutasteride (0.5 mg, Glaxo Smith Kline Pharmaceuticals, Poznań, Poland) or finasteride (5 mg; Gedeon Richter, Budapest, Hungary). Local ethical committee approval and informed consent were obtained.

### 3.9 Data analysis

Assay validation data are presented as mean (RSD) and biological concentrations as mean±standard error of the mean. Effects of drugs on concentrations of substrates and product of 5α-reductases were tested by repeated measure ANOVAs with Fisher׳s post-hoc tests and associations tested by Pearson Correlation. Where appropriate, data below the limits of quantitation were imputed as 0.5xLLOQ for statistical tests.

## 4 Results and discussion

### 4.1 Mass spectrometric conditions and fragmentation of analytes

All analytes and internal standards in solution ionised to form their protonated molecular ions in both positive electrospray (ESI) and APCI modes (Table 1). Although others have previously used ESI [24], APCI was selected due to less ion suppression during steroid analysis in the biomatrix. Signal responses in post-spiked serum compared to unextracted serum were androstenedione 110.8% (1.7%), testosterone 115.3% (2.1%), DHT 109.2% (2.4%), finasteride 111.0% (0.9%), dutasteride 97.2% (3.0%). APCI typically suffers less from ion suppression due to reduced ionisation of phospholipids and other competing compounds [35].

**Table 1:** Mass spectral conditions for analysis of analytes and internal standards utilising atmospheric pressure chemical ionisation.

	Mass (amu)	Precursor ion (*m*/*z*)	Product ion Quan; Qual	Declustering potential (V)	Collision energy (V) Quan; Qual	Cell exit potential (V) Quan; Qual
**ANALYTES**						
Androstenedione	286.4	287	97; 109	56	29; 27	18; 12
Testosterone	288.4	289	97; 109	16	27; 31	14; 16
5α-Dihydrotestosterone^⁎^	290.4	291	255; ^⁎^	16	21; ^⁎^	28; ^⁎^
Finasteride	372.5	373	317; 305	26	27; 41	14; 28
Dutasteride	528.5	529	461; 264	161	45; 55	48; 32

**Internal Standards**						
^13^C_3_-androstenedione	289.4	290	100; 112	31	27; 39	12; 16
^13^C_3_-testosterone	291.4	292	100; 112	1	27; 35	4; 8
^13^C_3_-dihydrotestosterone^⁎^	293.4	294	258: ^⁎^	61	21; ^⁎^	12; ^⁎^
d9-Finasteride	381.6	382	318; 314	41	33; 41	34; 36

Amu: atomic mass unit; Quan: quantifier ion; Qual: qualifier ion; V: volts. ^⁎^: Qualifier ion not detected in biological matrix.

Androgens fragmented as previously reported [24] to yield product ions incorporating the A-ring of the steroid [36] and the presence of the three ^13^C atoms was evident in the product ions of their internal standards. Finasteride, d9-finasteride and dutasteride fragmented as reported previously [15], [34]. It was important to enhance the signal for DHT, the least abundant analyte with poorest ionisation efficiency, hence source conditions were optimised for this analyte. Quantifier and qualifier transitions were defined for each analyte and internal standard (Table 1); however, in the case of DHT the signal from the potential qualifier ion (*m*/*z* 291→91) was not detectable in the biological matrix. The 3α, 5α-reduced product formed from androstenedione was included in initial screening but was not pursued into full validation. This steroid generated ions with similar intensity to DHT but, due to lower substrate concentrations, this product was present in concentrations which could not be readily detected in 500 μL of serum.

### 4.2 Selection of internal standards

^13^C labelled internal standards of androgens were selected, since some deuterium labelled steroids proved unsuitable due to variable loss of stable-isotopes during sample processing or ionisation, and isotopomers labelled in the D-ring did not retain deuterium in the product ion. Loss of deuteriums (particularly when attached to the steroid A-ring), has been described by others [37], [38], [39]. ^13^C_3_-Androstenedione, ^13^C_3_-testosterone and ^13^C_3_-DHT proved suitable for robust quantitation as described further, subject to chromatographic resolution. For example, the mass +1 isotopomer of ^13^C_3_-androstenedione could interfere with quantitation of DHT.

We have previously reported the synthesis of d9-finasteride [34] and others have used d3-finasteride as the internal standard for gas chromatography–MS [40]. In all other reports of finasteride and dutasteride analysis, non-deuterated internal standards were used, with finasteride often used as the internal standard for dutasteride [15], [16], and other compounds used for finasteride quantitation [18], [19], [20], [29]. d9-Finasteride had sufficient structural similarity to both 5α-reductase inhibitors for robust analysis of either drug, however synthesis of deuterated dutasteride in future may offer added benefits for precision and accuracy.

### 4.3 Chromatographic conditions

The potential for interference between endogenous positional isomers (testosterone and DHEA), stereoisomers (testosterone and its 17α-epimer) and also isobaric isotopomers from naturally occurring ^13^C_2_ isotopomers was noted, requiring selective chromatographic approaches. This combined approach had not been achieved previously for steroids and drugs. Reported methods for detection of individual analytes predominantly use C18 columns, though these varied in length from 50 mm [15], [16], [17] to 150 mm [19], [20]. Attempts with alternative stationary phases were not successful with all analytes. Although finasteride could be efficiently eluted with the steroids, combined analysis with dutasteride proved more challenging. Adequate peak resolution was seen with most analytes of interest using a pentafluorophenyl column, however dutasteride was not detected; with 6 fluorine atoms and an aromatic unit in the molecule, dutasteride may have a much greater affinity for the column, though *π*–*π* interactions. Greatest peak intensities for all analytes were found with a Kinetex C18 column, a porous shell column, and acceptable peak resolution with column length of 150 mm.

Acetonitrile has often been selected as the organic component of the mobile for analysis of dutasteride and finasteride [18], [19], [20], and methanol for androgens [24], [41]. However, acetonitrile suppressed ionisation of all analytes and hence methanol was selected. Ionisation was improved when formic acid was added as a modifier, with 0.1% yielding maximum responses, while still retaining consistency in chromatographic separation. We did not observe the improvements in analysis of testosterone, androstenedione and finasteride reported previously following buffering formic acid with ammonium acetate [17], [42]. The duration of the gradient was optimised, being the key component allowing optimal baseline separation (particularly of testosterone and epi-testosterone), ultimately requiring a 19 min run. Extension of the isocratic time after the initial gradient was important to maintain peak symmetry of DHT.

### 4.4 Extraction

Extraction was optimised to maximise recovery of endogenous DHT from serum, while extracting both androgens and 5α-reductase inhibitors. DHT and dutasteride proved the most challenging analytes.

Based on previously published reports, we evaluated a series of liquid-liquid extractions employing ethyl acetate:hexane (3:2, *v/v,* with and without NaOH (0.1 M)) and ethyl acetate (with and without saturated NaCl) with samples tested neat, mixed with water, acidified (mixed with 0.1% FA) or alkaline (mixed with 5% ammonia). Despite good recovery of testosterone and androstenedione, these approaches did not recover endogenous DHT or the 5α-reductase inhibitors efficiently. In supported liquid extractions, pre-extraction mixing was tested with FA (1%, 0.1%), water, NH_4_OH (0.1 M), and acetic acid (0.1%), and extracting solvents tested were dichloromethane, diethyl ether, ethyl acetate, hexane, methanol and acetonitrile. Supported liquid-liquid extractions proved highly variable for steroid analysis, and recoveries achieved for 5α-reductase inhibitors were inadequate. Protein precipitation and phospholipid crash methods gave poorer recoveries and again endogenous DHT was not detected. Solid-phase extraction had been used previously in separate assays for 5α-reductase inhibitors [15], [18] and androgens [26], [43] and the reversed-phase polymeric sorbent, Oasis® Hydrophilic Lipophilic Balanced (HLB), was ultimately selected. This technology also allowed transfer to 96-well plate format, suitable for high-throughput processing.

**Fig. 2:**
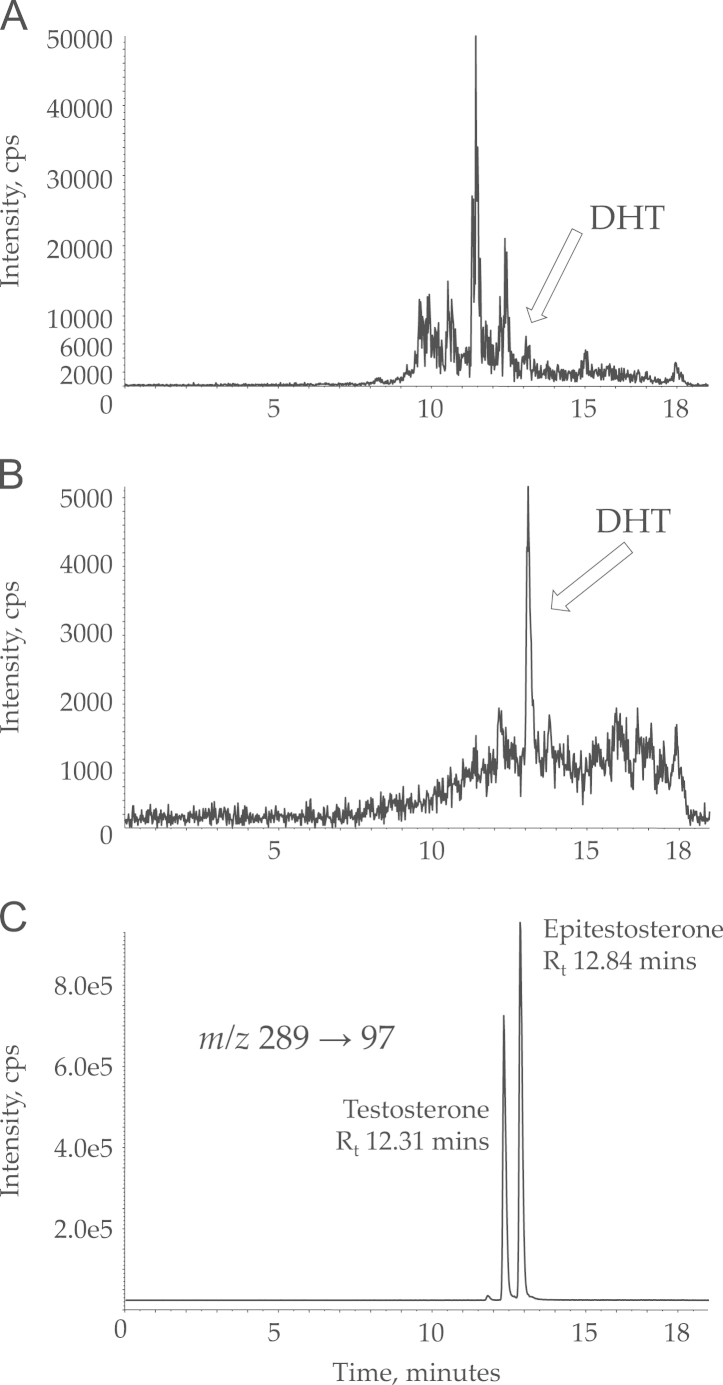
Mass chromatograms demonstrating analytical challenges. Representative mass chromatograms demonstrating improvement in signal to noise in mass transition (*m*/*z* 291→255) representing DHT recovered from normal male serum, following optimisation of the wash step. (A) Wash step of 5% methanol in water. (B) Wash step of 50% methanol in water. DHT, dihydrotestosterone; cps, counts per second. (C) Mass chromatogram (*m*/*z* 289→97) demonstrating separation of endogenous isomers of testosterone. cps, counts per second.

Mixing serum with water prior to extraction yielded the best extraction efficiency and the composition of the wash step proved vital to decrease background noise in the DHT transition. When using only 5% methanol in the wash step, several large peaks eluted close to the retention time of DHT; these could be eliminated by washing with 50% methanol in water, followed by elution in methanol (Fig. 2A and B). Using the final methods, extraction efficiencies all exceeded 80%: androstenedione 88.7% (15.4%); testosterone 84.6% (13.4%); DHT 85.5% (14.7%); finasteride 89.6% (14.3%); dutasteride 94.5% (10.4%).

## 5 Assay validation

### 5.1 Analyte specificity

All analytes and internal standards were temporally resolved from potential isomeric and isobaric interferences. Of note, testosterone and its biologically inert epi-isomer, epitestosterone, were separated chromatographically (Fig. 2C). The highly abundant isobaric steroid DHEA was not detected in the mass transitions (both quantifier and qualifier) monitored for testosterone. At amounts used, internal standards did not give any detectable interference in the analyte transitions.

An additional consideration was the potential presence of metabolites of 5α-reductase inhibitor drug in serum. As well as unchanged drug, dutasteride is known to have 3 major (4′-hydroxydutasteride, 1,2-dihydrodutasteride, and 6-hydroxydutasteride) and 2 minor (6,4′-dihydroxydutasteride and 15-hydroxydutasteride) metabolites detected in human serum following dosing to steady state [44]. Finasteride has two main in vivo metabolites detected in serum [45]. With different molecular weights to their parent drug, metabolites of both finasteride and dutasteride would be anticipated to give rise to different precursor ions and mass transitions and hence not be detected in the current assay.

### 5.2 LOD and LLOQ

Analyte limits of detection and lower limits of quantitation (Table 2) permitted analysis of anticipated concentrations of analytes in serum and were similar to those of Kulle et al. [26] for testosterone and androstenedione, although slightly poorer for DHT. The anticipated reference ranges for expected concentrations in serum in adult men are: androstenedione 0.23–2.41 ng/mL [23], [46], testosterone 2.65–9.71 ng/mL and DHT 0.14–0.77 ng/mL [24], finasteride 1.8–49 ng/mL [45] and dutasteride 36 ng/mL [44]. Notably finasteride and dutasteride could be quantified accurately even in amounts with signal:noise ≤3 (defined as the LOD). These limits would permit analysis of most analytes (except DHT) in volumes as low as 10 μL but to allow the incorporation of DHT, 500 μL of serum was required.

**Table 2:** Limits of detection and lower limits of quantitation.

	Standard curve range (ng/mL)	LOD (ng/mL)	LLOQ (ng/mL)
Androstenedione	0.1–5	0.08	0.125
Testosterone	1–15	0.003	0.005
5α-Dihydrotestosterone	0.1–5	0.13	0.21
Finasteride	1–100	0.02	0.003
Dutasteride	1–100	0.2	0.1

Abbreviations: LOD: limits of detection, LLOQ: lower limits of quantitation.

### 5.3 Linearity

Standard curves were linear in the range required, and for all analytes mean *r* was >0.99 (SD between 0.002–0.004). Mean intercepts (SD) were: androstenedione −0.007 (0.02); testosterone 0.084 (0.31); DHT−0.011 (0.02); finasteride 0.044 (0.06); dutasteride 8.13E^−08^ (2.42E^−07^). 1/*x* weighting was used for all analytes except DHT, where no weighting was applied.

### 5.4 Precision and accuracy

Intra- and inter-assay precision and accuracy of analysis are summarised in Table 3. While acceptable results were obtained for testosterone and androstenedione across the range of concentrations tested, increasing inter-assay variability in DHT analysis was observed at low values, close to the LLOQ. In the case of finasteride and dutasteride, acceptable intra- and inter-assay precision and accuracy were demonstrated across the range of concentrations anticipated in clinical studies (Table 3), although in future stable-isotope labelled dutasteride might allow for lowering of the LLOQ. Acceptable reproducibility upon repeat injections of standards and samples was demonstrated with relative standard deviations between 3.1 and 5.9%.

**Table 3:** Intra-assay and inter-assay precision and accuracy.

	Target concentration (ng/mL)	Intra-assay (*n*=6)			Inter-assay (*n*=4)		
		Concentration (ng/mL): mean (SD)	Precision (% RSD)	Accuracy (%)	Concentration (ng/mL): mean (SD)	Precision (% RSD)	Accuracy (%)
Androstenedione	Low (0.2)	0.18 (0.01)	5.4	87.6	0.19 (0.02)	19.3	93.6
	Mid (2)	1.65 (0.12)	7.2	101	1.75 (0.16)	8.9	92.3
	High (5)	4.22 (0.27)	6.5	98.3	5.01 (0.59)	11.9	103.4
	Sample	0.16 (0.02)	9.9	–	0.20 (0.04)	20.1	–

Testosterone	Low (2)	1.80 (0.07)	2.8	94.4	1.88 (0.19)	10.0	94.9
	Mid (7.5)	6.50 (0.42)	5.8	101	7.29 (0.83)	11.3	100.6
	High (15)	14.40 (0.75)	4.9	100	15.28 (0.65)	4.2	103.3
	Sample	9.40 (0.61)	6.0	–	4.22 (0.21)	5.0	–

5α-Dihydrotestosterone	Low (0.2)	0.17 (0.02)	11.8	87	0.23 (0.04)	15.5	119.5
	Mid (2)	1.70 (0.11)	6.4	90	1.83 (0.14)	7.4	100.0
	High (5)	4.11 (0.20)	4.9	103	5.00 (0.60)	12.1	105
	Sample	1.39 (0.19)	12.8	–	1.13 (0.31)	27.3	–

Finasteride	Low (2)	2.18 (0.24)	10	101	1.87 (0.30)	16.2	92.7
	Mid (25)	24.68 (1.34)	5.5	104	24.51 (2.08)	8.5	100.9
	High (100)	95.69 (6.23)	6.5	101	99.17 (4.35)	4.4	102.3
	Sample	8.45 (0.35)	4.1	–	8.96 (0.69)	7.8	–

Dutasteride	Low (2)	1.67 (0.37)	22	88.2	1.86 (0.16)	8.8	94.5
	Mid (25)	25.07 (2.40)	9.6	110	26.00 (2.60)	10.0	107.4
	High (100)	107.51 (16.06)	14.9	108	86.5 (4.40)	5.1	105
	Sample	16.25 (1.83)	11.2	–	15.05 (1.26)	8.4	–

### 5.5 Stability

Acceptable autosampler and extract storage stability were demonstrated for a calibration curve and patient samples, as shown in Table 4, with less than 10% decline during typical handling conditions.

**Table 4:** Stability upon storage.

	Relative response after 24 h in autosampler (10 °C)	Relative response after 28 days in freezer (−20 °C)
Androstenedione	102.7%	98.3%
Testosterone	99.7%	101.0%
5α-Dihydrotestosterone	90.0%	97.5%
Finasteride	95.3%	103.4%
Dutasteride	92.0%	92.0%

Relative response for calculated concentrations for all analytes in a single patient sample after 24 h in the autosampler (10 °C) and after 28 days in the freezer (−20 °C).

### 5.6 Pharmacodynamic assessments in clinical samples

**Fig. 3:**
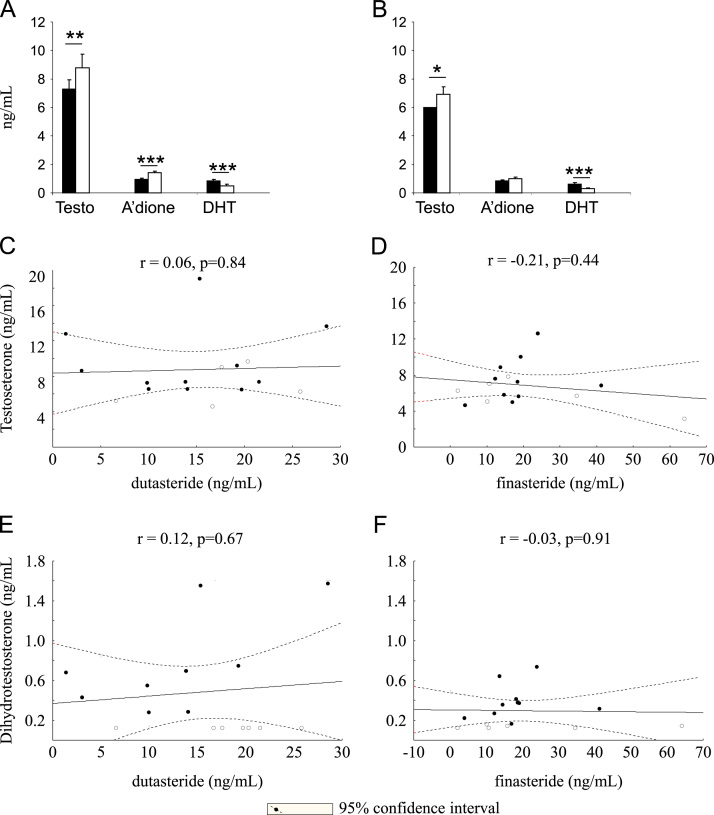
(A and B) The concentrations of testosterone (Testo) and androstenedione (A׳dione) were significantly increased by dutasteride and those of testosterone only by finasteride. Dihydrotestosterone (DHT) concentrations were reduced by both drugs. Data are mean±SEM (*n*=16), compared by repeated measure ANOVA, with Fisher׳s post-hoc test. Correlations were not observed between the concentrations of testosterone (C; dutasteride: D; finasteride) or DHT (E; dutasteride: F; finasteride) and those achieved of drug. Data show Pearson correlations, depicted with 95% confidence intervals. Date points (for DHT) which were recorded as less than the limit of detection were imputed at 0.125 ng/mL for statistical purposes and are represented in open circles.

The assay presented was applied to a clinical research study with male volunteers studied prior to and following three months of finasteride (5 mg daily) or dutasteride (0.5 mg daily) [12]. Dutasteride and finasteride treatment resulted in a 46.5% and 47.1% suppression of DHT concentrations respectively (Fig. 3A and B). This is somewhat less than reported previously [47], but provided a robust marker of target engagement. The concentrations of dutasteride achieved would be anticipated to inhibit both isozymes of 5α-reducatse effectively. Relationships were not observed between amounts of DHT and those of dutasteride or finasteride, possibly due to maximal inhibition of the enzyme (Fig. 3C and D); doses of finasteride greater than 5 mg/day do not to achieve an increase in efficacy [48] and likewise the concentrations achieved of dutasteride were close to the maximal effect demonstrated by Gisleskog et al. [49]. The reduction in DHT was accompanied by an increase in the concentrations of the enzyme substrate, testosterone, following treatment with both finasteride and dutasteride, of a similar magnitude to previously reported [50]. Androstenedione, a further substrate also increased significantly following dutasteride treatment, with a strong trend evident with finasteride (*p*=0.06).

With pre-treatment concentrations of DHT approximately 0.8 ng/mL, it is anticipated that measurement of suppression of DHT to ~25% of its original value would be possible using this analytical approach. Levels fell below the LLOQ in more subjects receiving dutasteride than finasteride. Increased sample or injection volume, derivatisation or further advances in technology may allow extension of the pharmacodynamic range measurable.

## 6 Conclusions

The novel method developed was suitable for simultaneous measurement of androgens, dutasteride and finasteride from human serum, despite significant challenges in chromatographic and extraction method development. The assay requires relatively little sample volume (500 µL), has a simple extraction method compatible with a 96-well format, and is able to quantify DHT without derivatisation, although advances in sensitivity would still be beneficial and allow quantitation of DHT and androstane-3α,5α-dione in smaller volumes. Expected concentrations of all analytes fell within the linear range of the standard curve in healthy men. The method is able to quantify DHT suppression to approximately 25% of normal values and can be used detect enzyme inhibition and compliance with both 5α-redcutase inhibitors. Intra- and inter-day precision and accuracy were acceptable and stability testing demonstrated the assay to be applicable to normal laboratory practice.
